# CT ventriculographic identification and neuroendoscopic treatment of acquired hydrocephalus due to cerebral aqueduct lesions: a two-case report

**DOI:** 10.3389/fonc.2025.1700195

**Published:** 2026-02-04

**Authors:** Kai Liu, Yuheng Feng, Shuli Wang, Yubo Zhang, Xiaoxuan Ji, Qiang Li, Xiaodong Luo

**Affiliations:** 1 Department of Neurosurgery, The Second Hospital of Lanzhou University, Lanzhou, Gansu, China; 2 The Second Clinical Medical School of Lanzhou University, Lanzhou, Gansu, China; 3 Jining No.2 People’s Hospital, Jining, Shandong, China

**Keywords:** aqueductal lesions, CT ventriculography, magnetic resonance imaging, neuroendoscopic resection, obstructive hydrocephalus

## Abstract

Hydrocephalus secondary to cerebral aqueduct lesions poses significant diagnostic and therapeutic challenges, particularly when causative lesions are occult on conventional neuroimaging. We present two pediatric cases of acquired obstructive hydrocephalus, in which routine laboratory tests were unremarkable except for mildly elevated tumor markers. Both patients exhibited acute symptoms of intracranial hypertension. Magnetic resonance imaging (MRI) demonstrated supratentorial hydrocephalus with periventricular edema but failed to visualize a definitive obstructive lesion. Subsequent cerebral ventriculography revealed aqueductal filling defects located at the aqueductal inlet along the floor of the third ventricle. Neuroendoscopic resection of the intraventricular lesions was successfully performed through a right frontal transventricular approach. Histopathological examination played a pivotal role in management: Both cases were postoperatively diagnosed as mixed germ cell tumors. Case 1 received postoperative chemoradiotherapy, whereas Case 2 was treated with postoperative chemotherapy alone. Both patients achieved rapid and sustained symptom relief, with no postoperative complications or recurrence observed during the 12-month follow-up period. These cases underscore the diagnostic value of ventriculography in identifying occult aqueductal lesions and highlight the dual diagnostic and therapeutic utility of neuroendoscopic resection, which provides a definitive histological diagnosis and restores cerebrospinal fluid circulation.

## Introduction

Hydrocephalus is a neurological disorder characterized by the pathological accumulation of cerebrospinal fluid (CSF) within the ventricular system, resulting in ventricular dilation and elevated intracranial pressure (1–3). Although congenital hydrocephalus is well documented, acquired hydrocephalus due to intraventricular space-occupying lesions of the cerebral aqueduct is rare and presents significant diagnostic challenges (4, 5). Magnetic resonance imaging (MRI) is the modality of choice for evaluating hydrocephalus; however, in some cases, the responsible lesion may not be clearly visualized, complicating clinical decision-making (6). Although less commonly employed in contemporary practice, cerebral ventriculography can provide valuable diagnostic information in selected patients when conventional neuroimaging fails to identify the obstructive pathology (7). Neuroendoscopy has emerged as a safe and effective minimally invasive technique for both the diagnosis and treatment of deep-seated intraventricular lesions, particularly those located in critical regions such as the cerebral aqueduct (7). Compared with palliative procedures such as ventriculoperitoneal shunting, neuroendoscopic resection of the obstructive lesion can restore normal CSF circulation, relieve clinical symptoms, and prevent shunt-related complications, thereby improving long-term outcomes and quality of life (8). Crucially, this approach also yields essential tissue for histopathological diagnosis, which is vital for guiding subsequent management (9). Here, we present two rare cases of acquired hydrocephalus secondary to cerebral aqueduct lesions that were occult on preoperative MRI but subsequently identified using cerebral ventriculography. Both patients underwent successful neuroendoscopic resection of the lesions, resulting in marked symptomatic improvement and favorable clinical outcomes. These cases underscore the diagnostic utility of ventriculography in selected patients and demonstrate the diagnostic and therapeutic utility of neuroendoscopic resection in managing aqueductal obstruction.

## Imaging evaluation

### MRI protocol

All patients underwent MRI using a 3.0-T scanner. The standard protocol comprised T1-weighted and T2-weighted imaging in axial, sagittal, and coronal planes, along with axial fluid-attenuated inversion recovery (FLAIR), diffusion-weighted imaging (DWI), and multiplanar contrast-enhanced T1-weighted imaging. Notably, advanced MRI techniques—such as high-resolution 3D T2-weighted imaging or phase-contrast cine MRI for CSF flow dynamics—were not included in the initial emergent protocol. The lack of these high-resolution, flow-sensitive sequences likely limited the sensitivity for detecting minute aqueductal lesions preoperatively.

### CT ventriculography protocol

CT ventriculography was performed using a nonionic iodinated contrast medium (Iohexol, 300 mg I/mL). Under strict aseptic conditions, a total volume of 3–5 mL of contrast was manually infused at a controlled rate via the external ventricular drainage (EVD) catheter. Immediate image acquisition was conducted using a multi-detector CT scanner. High-resolution reconstruction was performed with a slice thickness of 0.625 mm. Multiplanar reformations (axial, coronal, and sagittal) were generated to meticulously delineate the anatomical configuration of the cerebral aqueduct and identify any intraventricular filling defects.

## Case report

**Figure 1:**
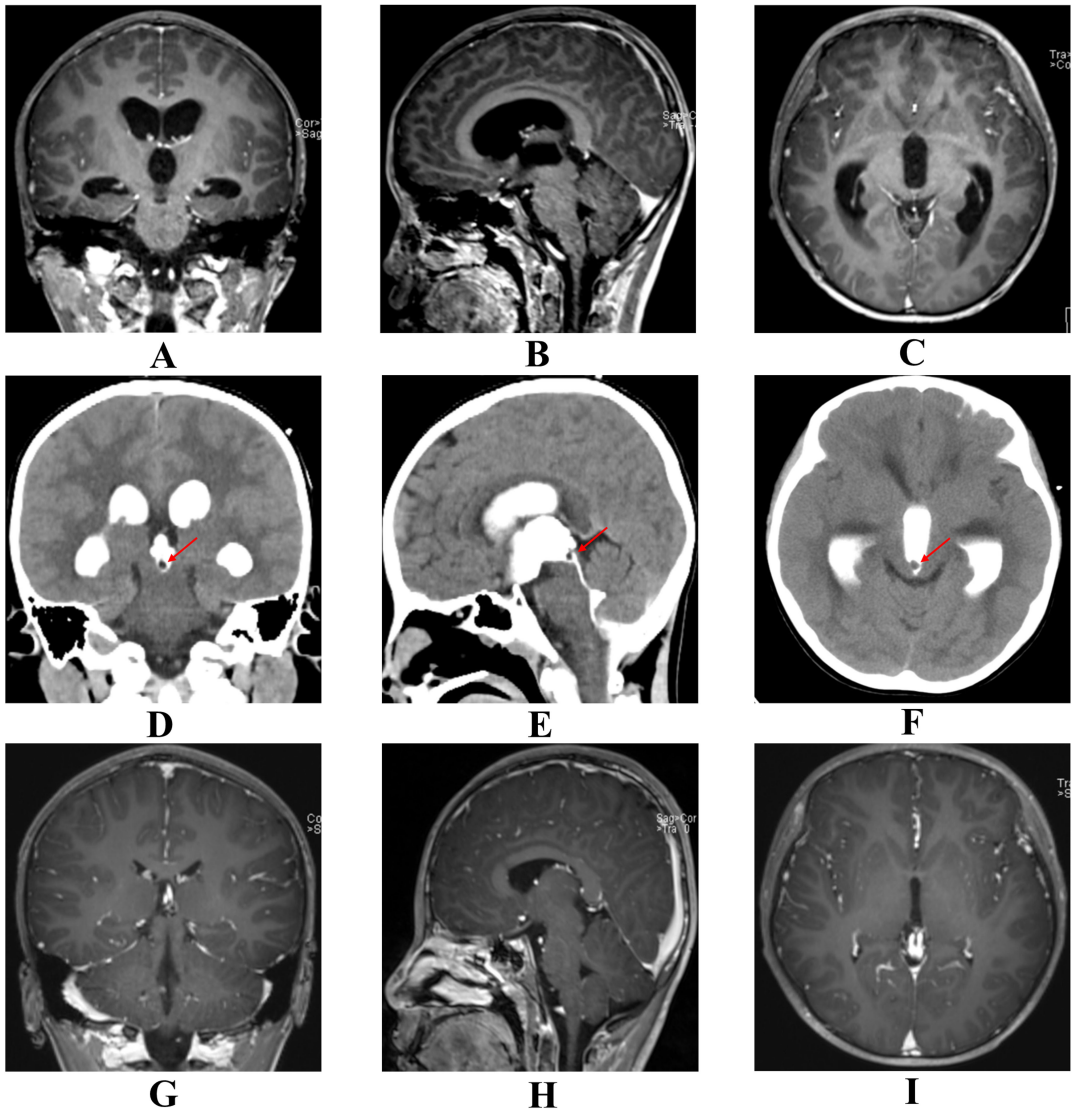
Pre- and postoperative radiological comparisons of Case 1. Images are aligned in consistent anatomical planes (Left column: Coronal; Middle column: Sagittal; Right column: Axial) to facilitate direct comparison. **(A–C)** Preoperative contrast-enhanced MRI demonstrating supratentorial ventriculomegaly. Note the absence of a clearly identifiable aqueductal lesion on these standard sequences. **(D–F)** Preoperative CT ventriculography revealing a 0.4-cm intraluminal filling defect at the aqueductal inlet along the floor of the third ventricle (arrows), clearly identifying the occult obstruction that was not visible on MRI. **(G–I)** Postoperative contrast-enhanced MRI at 72 hours showing a significant reduction in ventricular size and confirming patency of the cerebral aqueduct, indicating successful resolution of hydrocephalus. Panels **(A, D)** are adapted with permission from Li et al. (32), Pineal germinoma diagnosed by iohexol CT ventriculography, Pediatric Radiology, (2025) 55(2):359, Springer Nature. This content is licensed by Springer Nature Customer Service Center GmbH (SNCSC) and is NOT part of the overriding OA/Creative Commons license of this publication. Reproduced with permission from SNCSC.

**Figure 2:**
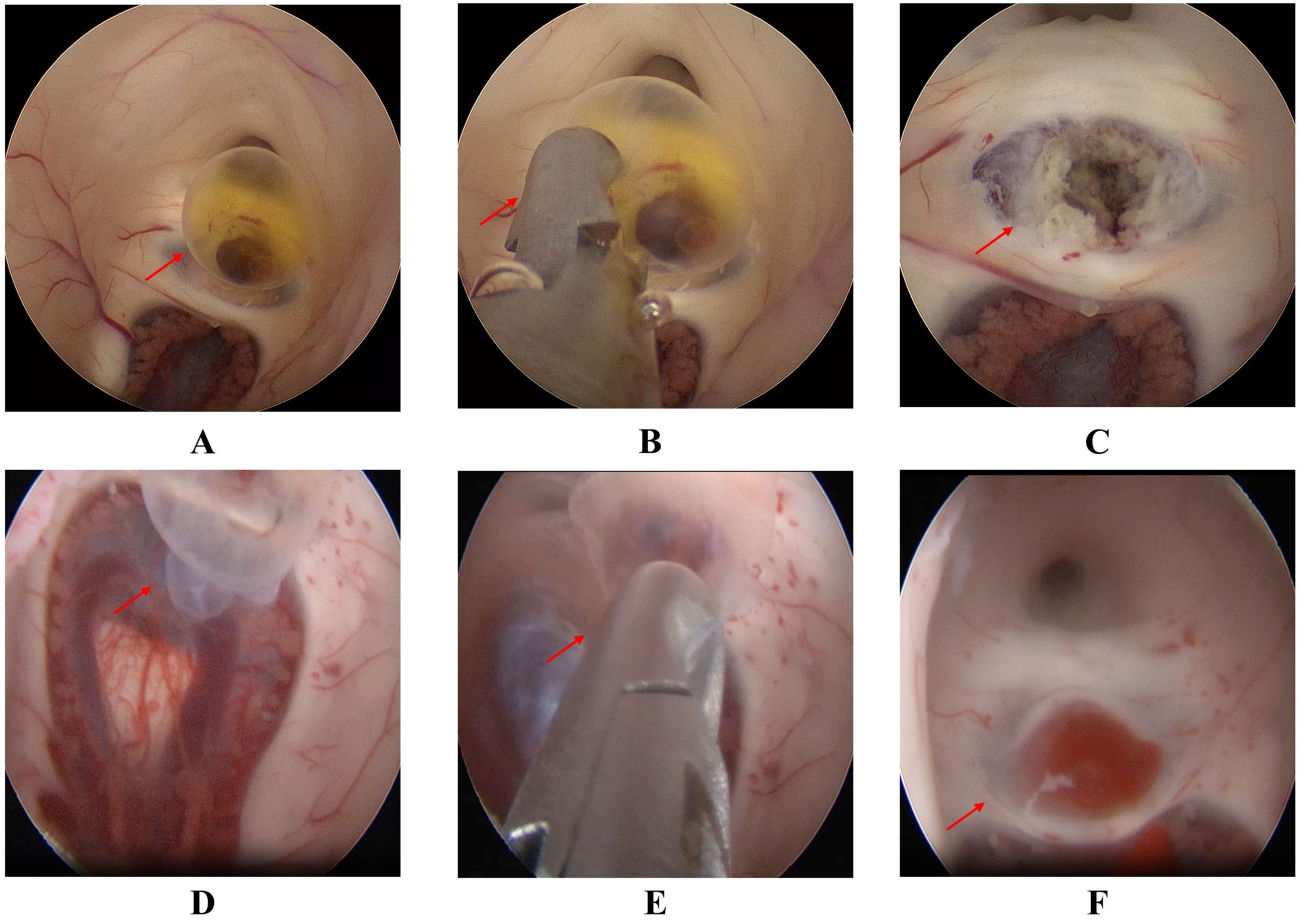
Intraoperative findings during neuroendoscopic resection. **(A–C)** Case 1: **(A)** Endoscopic visualization of the cystic-solid lesion obstructing the aqueductal inlet (arrow). **(B)** Resection of the cyst wall using endoscopic forceps (arrow). **(C)** Restoration of aqueductal patency after gross total resection (arrow). **(D–F)** Case 2: **(D)** Endoscopic visualization of the vascularized cystic mass at the aqueductal inlet (arrow). **(E)** Careful dissection and removal of the tumor (arrow). **(F)** Verified patency of the aqueduct following lesion removal (arrow).

Case 1 was a boy aged 9 years and 4 months who was admitted in October 2023. He presented with a 3-day history of intermittent frontal headache occurring 3–5 times per day, which intensified the day before admission. Profuse vomiting developed approximately 12 hours prior to hospitalization. On neurological examination, the patient was alert and oriented. Cranial nerve function was normal, and motor and sensory examinations were unremarkable. There was no gait ataxia or ocular motility restriction. Signs of meningeal irritation and papilledema were absent. Preoperative AFP was 22.30 ng/mL and HCG was <1.00 mIU/L. All other examinations, including routine blood tests, liver and kidney function tests, and assessments of immune and coagulation function, were within normal limits. The patient had no history of trauma or genetic disorders and was born at full term with an unremarkable perinatal history. MRI revealed supratentorial ventricular enlargement with hydrocephalus and bilateral periventricular edema, but no definitive causative lesion was identified (**Figures 1A–C**). Subsequent ventriculography revealed a 0.4-cm filling defect located at the aqueductal inlet along the floor of the third ventricle (**Figures 1D–F**). The patient underwent neuroendoscopic resection of the lesion via a right frontal transventricular approach. Upon endoscopic entry into the posterior third ventricle, the lesion appeared as a cystic-solid mass compressing the aqueduct (**Figure 2A**). The cyst wall exhibited irregular papillary-like projections with a relatively rich vascular supply and focal bleeding. The cystic component contained yellowish fluid that partially obstructed the aqueduct. Both cystic and solid portions were carefully resected with endoscopic forceps, achieving near-total removal under direct visualization (**Figures 2B, C**). Following resection, the aqueductal inlet was confirmed to be patent. Histopathological examination of the specimen confirmed the diagnosis of a mixed germ cell tumor exhibiting benign histological features without cellular atypia, with immunohistochemistry (IHC) showing Vimentin (+), SSTR (+), CD99 (+), CD56 (partial+); CK (-), EMA (-), Olig-2 (-), Fli-1 (-), Syn (-), NSE (-), PLAP (-), CD117 (-), SALL4 (-), OCT3/4 (-); and Ki-67 (20%). However, the high Ki-67 index and the elevated preoperative serum AFP level (22.30 ng/mL) were inconsistent with a benign cyst. Consequently, a secondary pathological consultation was sought, which revised the diagnosis to a mixed germ cell tumor. Subsequently, the patient underwent postoperative craniospinal irradiation and chemotherapy with the VIP regimen (etoposide, ifosfamide, and cisplatin). Postoperative MRI demonstrated a significant reduction in the ventricular size (**Figures 1G–I**), with serum AFP 10.70 ng/mL and HCG <1.00 mIU/L. At the one-year postoperative review, both imaging and tumor markers remained normal, and the patient was neurologically intact, asymptomatic, and fully resumed normal daily and school activities with preserved quality of life.

**Figure 3:**
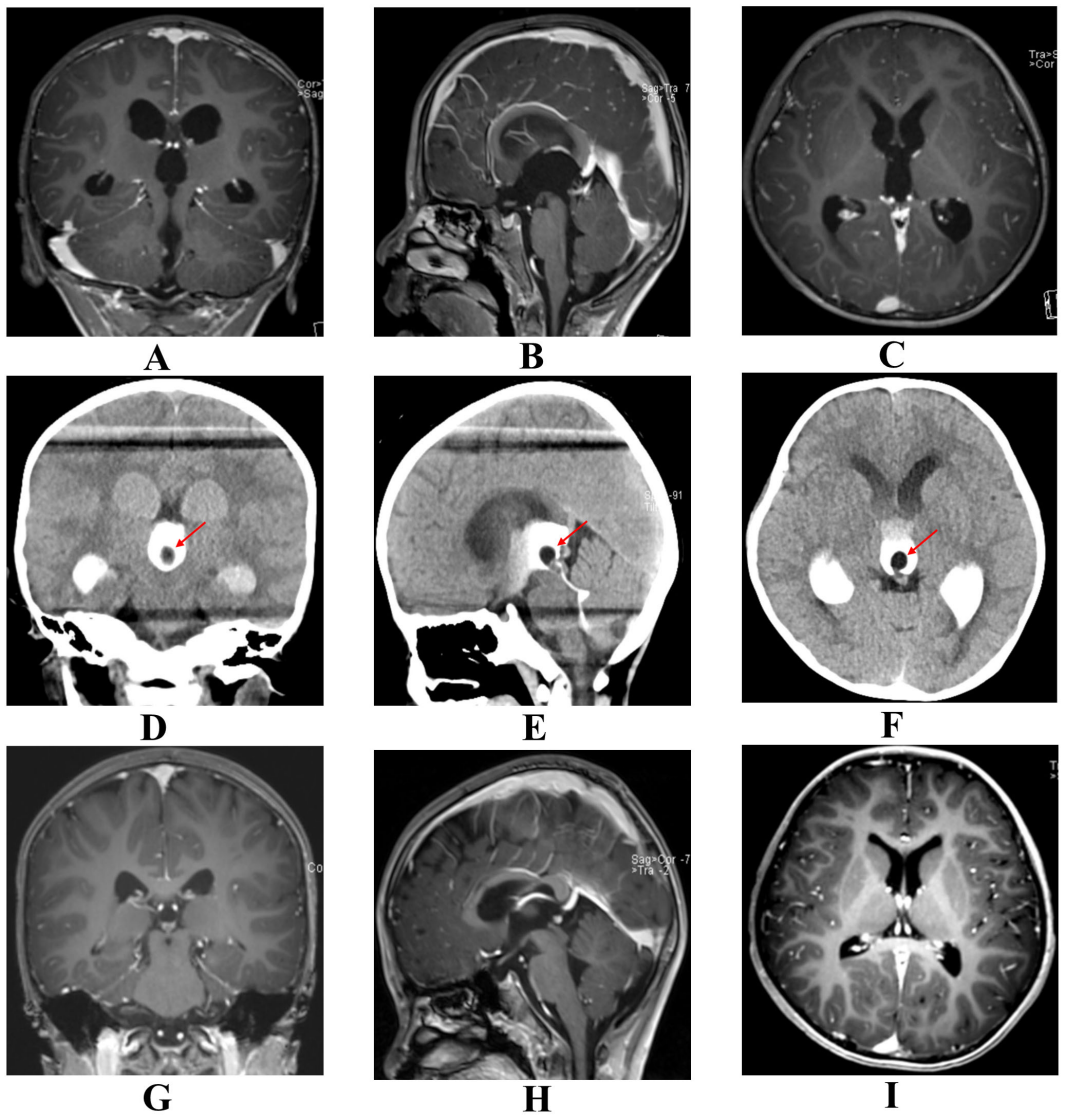
Pre- and postoperative radiological comparisons of Case 2. Images are aligned in consistent anatomical planes (Left column: Coronal; Middle column: Sagittal; Right column: Axial) to facilitate direct comparison. **(A–C)** Preoperative MRI showing supratentorial hydrocephalus with a funnel-shaped aqueduct but no definitive mass. **(D–F)** Preoperative CT ventriculography revealing a 0.8-cm filling defect at the aqueductal inlet (arrows), which identified the occult obstruction. **(G–I)** Postoperative contrast-enhanced MRI at 72 hours showing a significant reduction in ventricular size and patent CSF pathways, confirming successful treatment.

Case 2 was a boy aged 3 years and 2 months who was admitted in August 2022 with a 3-day history of recurrent vomiting, nausea, and intermittent abdominal discomfort. The vomiting was non-bilious and non-bloody, with a frequency exceeding 10 episodes per day. After gastrointestinal etiology was excluded by a gastroenterologist, a neurological cause was suspected. The neurological examination was limited by the patient’s poor cooperation; however, the Babinski sign was noted to be equivocal. Preoperative AFP was 14.00 ng/mL and HCG was 8.27 mIU/L, while other hematological, biochemical, immunological, and coagulation tests were within normal ranges. The patient had no history of trauma or hereditary disorders and was born at full term, with an unremarkable perinatal history. MRI revealed supratentorial hydrocephalus, with the aqueduct demonstrating a funnel-shaped configuration and possible adhesion at the inferior margin (**Figures 3A–C**), although no definite obstructive lesion was identified. Subsequent ventriculography demonstrated a 0.8-cm filling defect at the aqueductal inlet adjacent to the third ventricular floor (**Figures 3D–F**). The patient underwent neuroendoscopic resection of the lesion via a right frontal transventricular approach. Upon endoscopic entry into the posterior third ventricle, a cystic mass was identified at the aqueductal inlet near the third ventricular floor. The lesion showed visible vascularity and focal hemorrhage, partially occupying the posterior third ventricle and closely associated with the internal cerebral veins (**Figure 2D**). Under direct endoscopic visualization, the cystic component was meticulously removed with endoscopic forceps within the operable field, while carefully preserving adjacent neurovascular structures. Near-total resection was achieved, and the aqueductal inlet was confirmed to be patent (**Figures 2E, F**). Postoperative histopathological examination initially suggested a mature teratoma, with IHC showing CK8/18(+), CKP (+), Syn (glial cells+), and LCA (lymphocytes+). However, the Ki-67 labeling index was 20%, indicating high proliferative activity. Due to this finding, a secondary pathological consultation was sought, which revised the diagnosis to a mixed germ cell tumor. Consequently, to address the malignant potential, the patient underwent adjuvant chemotherapy with the VIP regimen. Considering the patient's young age (3 years and 2 months), radiotherapy was omitted to minimize the risk of long-term neurocognitive and developmental toxicity. Postoperative MRI demonstrated a significant reduction in the ventricular size (**Figures 3G–I**), with serum AFP 8.71 ng/mL and HCG <0.40 mIU/L. Over the course of the 12-month surveillance, the patient completed adjuvant chemotherapy at a local hospital without adverse events. Follow-up imaging and tumor markers remained within normal limits. Clinically, the patient remained asymptomatic and successfully returned to age-appropriate daily activities with no neurological deficits.

The detailed clinical characteristics, imaging findings, surgical data, and follow-up outcomes of the two patients are summarized in **Table 1**.

**Table 1:** Summary of clinical characteristics, management, and outcomes of the two patients.

Variable	Case 1	Case 2
Age	9 years and 4 months	3 years and 2 months
Sex	Male	Male
Chief complaint	Intermittent headache (3 days) and acute vomiting (12 hours)	Recurrent vomiting and nausea (3 days)
MRI	Supratentorial hydrocephalus with periventricular edema; no definitive lesion	Supratentorial hydrocephalus; funnel-shaped aqueduct with suspected adhesion, yet no definite obstructive lesion
CT ventriculography	Filling defect (0.4 cm) at the aqueductal inlet	Filling defect (0.8 cm) at the aqueductal inlet
ICP (mmH_2_O)	Not measured*	Not measured*
Surgical procedure	Neuroendoscopic resection	Neuroendoscopic resection
Surgical complications	None	None
Pathological diagnosis	Mixed germ cell tumor	Mixed germ cell tumor
Adjuvant radiotherapy	Craniospinal irradiation	None
Adjuvant chemotherapy	VIP regimen	VIP regimen
Follow-up duration (months)	12	12
Outcome at last follow-up	Asymptomatic; no recurrence	Clinically stable; no progression

VIP, etoposide, ifosfamide, and cisplatin; MRI, magnetic resonance imaging; CT, computed tomography; ICP, intracranial pressure.

*Intracranial pressure was not measured via lumbar puncture as it was contraindicated in the presence of severe obstructive hydrocephalus due to the risk of brain herniation.

Written informed consent for the publication of the patients’ clinical details and accompanying images was obtained from their legal guardians.

## Discussion

In pediatric secondary obstructive hydrocephalus, when routine neuroimaging fails to reveal a causative lesion, cerebral ventriculography may be required to localize the obstruction and guide management (10). Among pineal region tumors, germ cell tumors are the most common, and predominantly cystic lesions are often difficult to detect by conventional imaging in the early stages; however, they may obstruct the aqueductal inlet and thus present with hydrocephalus as the initial manifestation (11–13). Some patients may develop “paroxysmal/episodic hydrocephalus,” characterized by recurrent headache, transient disturbances of consciousness, and vomiting, with MRI findings ranging from negative to mild or inconsistent ventricular changes (14). This phenomenon is attributed to dynamic changes in cyst size, relative mobility of the lesion due to CSF dynamics and body position, and progressive ventricular dilatation, which intermittently relieve or reestablish aqueductal obstruction (15, 16).

The present report illustrates two rare cases of acquired hydrocephalus caused by cerebral aqueduct space-occupying lesions that were not clearly visualized on conventional MRI but were successfully identified through CT ventriculography. These findings emphasize the diagnostic value of this technique when noninvasive neuroimaging yields inconclusive results (17). Although ventriculography has been largely replaced by MRI in routine practice, its ability to directly delineate intraventricular filling defects remains clinically relevant (18). While advanced MRI sequences, such as high-resolution 3D T2-weighted imaging and phase-contrast cine MRI, offer superior assessment of CSF flow and anatomical detail, their clinical utility in symptomatic pediatric patients is often limited by long acquisition times and susceptibility to motion artifacts (19, 20). In the present cases, the acute presentation and potential risks of prolonged sedation precluded these lengthy protocols. Regarding the missed diagnoses on standard MRI, several factors likely contributed to the false-negative findings. First, the rapid, pulsatile flow of CSF within the narrow cerebral aqueduct creates flow void artifacts, which can mask small intraluminal lesions (21). Second, the obstructive membranes or micro-cysts were extremely thin and isointense to CSF, rendering them indistinguishable on standard sequences due to partial volume averaging (22). Consequently, CT ventriculography served as a rapid and decisive problem-solving tool (23). Unlike static MRI, it provided a functional assessment of aqueductal patency, clearly delineating the filling defect to confirm the obstruction (23). As fast-acquisition MRI technologies evolve, these noninvasive techniques are expected to become the standard alternative in the future (24).

Furthermore, neuroendoscopic resection of the obstructive lesions provided substantial symptomatic relief and favorable long-term outcomes, with no recurrence observed during follow-up (25–27). In contrast to ventriculoperitoneal shunting, which is associated with long-term hardware-related complications and shunt dependency, or endoscopic third ventriculostomy (ETV), which bypasses the obstruction but leaves the lesion *in situ*, neuroendoscopic resection provides a definitive anatomical solution (22, 28). Given that the obstruction in our cases was caused by discrete intraluminal masses, this targeted approach was considered the optimal strategy. It re-establishes physiological CSF circulation and avoids potential cyst regrowth or tumor progression—limitations inherent to non-resective techniques—while securing essential tissue for histopathological diagnosis, which proved critical for guiding adjuvant therapy in both cases. Despite these advantages, both CT ventriculography and neuroendoscopic resection carry inherent procedural risks. Ventriculography is invasive and may lead to complications such as infection, hemorrhage, or contrast-related adverse reactions (29). Similarly, neuroendoscopic resection in this region presents unique technical challenges due to the narrow working corridor of the aqueduct and the proximity to the eloquent periaqueductal gray matter (30). Risks include transient neurological deficits, oculomotor palsies, or memory disturbances (30). Therefore, patient selection must be precise: candidates are defined as those with symptomatic hydrocephalus and a radiologically confirmed focal, resectable obstruction at the aqueductal inlet. While MRI is the primary imaging modality, CT ventriculography serves as the critical selection tool for cases where clinical suspicion is high but standard MRI findings are inconclusive. To ensure safety, the use of a flexible neuroendoscope and meticulous hemostasis are essential to prevent injury to adjacent midbrain structures (31).

Several case-specific limitations should be acknowledged. First, the diagnostic process for both cases highlights the inherent challenges of intracranial germ cell tumors, where clinical markers and pathological findings must be carefully integrated to achieve an accurate diagnosis. Finally, although follow-up in both patients extends to approximately 12 months with no clinical or radiographic recurrence observed, this duration is still relatively short. Therefore, longer-term surveillance remains necessary to definitively rule out late recurrence.

Taken together, these cases highlight the importance of a stepwise diagnostic approach integrating ventriculography when MRI is inconclusive and demonstrate the diagnostic and therapeutic utility of neuroendoscopic resection. Future studies with larger patient cohorts are warranted to validate these findings, refine patient selection criteria, and further establish the role of ventriculography and neuroendoscopic resection in the management of aqueductal hydrocephalus.

## Conclusion

In summary, we report two rare cases of acquired hydrocephalus secondary to cerebral aqueduct space-occupying lesions that were occult on standard MRI but successfully identified through CT ventriculography. Neuroendoscopic resection not only restored physiological cerebrospinal fluid circulation with favorable long-term outcomes but also yielded essential tissue for histopathological diagnosis, which is critical for guiding subsequent management. These findings underscore the diagnostic utility of CT ventriculography in selected diagnostic dilemmas and highlight neuroendoscopic resection as a safe and effective therapeutic option for these challenging cases.

## Data Availability

The original contributions presented in the study are included in the article/supplementary material. Further inquiries can be directed to the corresponding author.

## References

[1] Hladky SB Barrand MA . Regulation of brain fluid volumes and pressures: basic principles, intracranial hypertension, ventriculomegaly and hydrocephalus. Fluids Barriers CNS. (2024) 21:57. doi: 10.1186/s12987-024-00532-w, PMID: 39020364 PMC11253534

[2] Kahle KT Kulkarni AV Limbrick DD Jr. Warf BC . Hydrocephalus in children. Lancet. (2016) 387:788–99. doi: 10.1016/s0140-6736(15)60694-8, PMID: 26256071

[3] Kahle KT Klinge PM Koschnitzky JE Kulkarni AV MacAulay N Robinson S . Paediatric hydrocephalus. Nat Rev Dis Primers. (2024) 10:35. doi: 10.1038/s41572-024-00519-9, PMID: 38755194 PMC12091269

[4] Dewan MC Rattani A Mekary R Glancz LJ Yunusa I Baticulon RE . Global hydrocephalus epidemiology and incidence: systematic review and meta-analysis. J Neurosurg. (2019) 130:1065–79. doi: 10.3171/2017.10.Jns17439, PMID: 29701543

[5] Sakata K Hashimoto A Kotaki Y Yoshitake H Shimokawa S Komaki S . Successful treatment of pure aqueductal pilomyxoid astrocytoma and arrested hydrocephalus with endoscopic tumor resection followed by chemotherapy: A case report and technical considerations. Neurosurg Pract. (2023) 4:e00030. doi: 10.1227/neuprac.0000000000000030, PMID: 39959717 PMC11809950

[6] Farb R Rovira A . Hydrocephalus and CSF disorders. In: Hodler J Kubik-Huch RA von Schulthess GK , editors. Diseases of the Brain, Head and Neck, Spine 2020-2023: Diagnostic Imaging. IDKD Springer Series: Switzerland: Springer (2020). p. 11–24., PMID:

[7] Gabbita AC Raju S . Management of complex hydrocephalus. Neurol India. (2021) 69:S350–s356. doi: 10.4103/0028-3886.332284, PMID: 35102987

[8] Hochstetler A Raskin J Blazer-Yost BL . Hydrocephalus: historical analysis and considerations for treatment. Eur J Med Res. (2022) 27:168. doi: 10.1186/s40001-022-00798-6, PMID: 36050779 PMC9434947

[9] Birski M Furtak J Krystkiewicz K Birska J Zielinska K Sokal P . Endoscopic versus stereotactic biopsies of intracranial lesions involving the ventricles. Neurosurg Rev. (2021) 44:1721–7. doi: 10.1007/s10143-020-01371-7, PMID: 32827050 PMC8121744

[10] Muñoz A Hinojosa J Esparza J . Cisternography and ventriculography gadopentate dimeglumine-enhanced MR imaging in pediatric patients: preliminary report. AJNR Am J Neuroradiol. (2007) 28:889–94., PMID: 17494664 PMC8134319

[11] Fakhran S Escott EJ . Pineocytoma mimicking a pineal cyst on imaging: true diagnostic dilemma or a case of incomplete imaging? AJNR Am J Neuroradiol. (2008) 29:159–63. doi: 10.3174/ajnr.A0750, PMID: 17925371 PMC8119086

[12] Fang AS Meyers SP . Magnetic resonance imaging of pineal region tumours. Insights Imaging. (2013) 4:369–82. doi: 10.1007/s13244-013-0248-6, PMID: 23640020 PMC3675249

[13] Vuong HG Ngo TNM Dunn IF . Incidence, prognostic factors, and survival trend in pineal gland tumors: A population-based analysis. Front Oncol. (2021) 11:780173. doi: 10.3389/fonc.2021.780173, PMID: 34869031 PMC8639690

[14] Guadix SW . Defining occult high−Risk cysts of the pineal region: A case series. Operative Neurosurgery. (2023) 24:572–81. doi: 10.1227/ons.0000000000000620, PMID: 36716050

[15] Ludwig HC Dreha-Kulaczewski S Bock HC . Paediatric pineal region cysts: enigma or impaired neurofluid system? Childs Nerv Syst. (2023) 39:3457–66. doi: 10.1007/s00381-023-06000-4, PMID: 37261536 PMC10684616

[16] Matsuzaki R Noguchi Y Aoki Y Oishi H Sugo N . A case of endoscopic treatment of a symptomatic pineal cyst. MOJ Clin Med Case Rep. (2023) 13:9–11. doi: 10.15406/mojcr.2023.13.00426

[17] Vankipuram S Srivastava C Ojha BK Chandra A Singh SK Jaiswal S . Management of multiloculated hydrocephalus in children with emphasis on role of CT ventriculography. Childs Nerv Syst. (2020) 36:2741–8. doi: 10.1007/s00381-020-04572-z, PMID: 32185473

[18] Muroi A Quezada JJ McComb JG . Usefulness of postoperative ventriculography and intracranial pressure monitoring following endoscopic third ventriculostomy. Childs Nerv Syst. (2021) 37:1151–8. doi: 10.1007/s00381-020-04981-0, PMID: 33241438

[19] Kraus MS Coblentz AC Deshpande VS Peeters JM Itriago-Leon PM Chavhan GB . State-of-the-art magnetic resonance imaging sequences for pediatric body imaging. Pediatr Radiol. (2023) 53:1285–99. doi: 10.1007/s00247-022-05528-y, PMID: 36255456

[20] Gräfe D Anders R Frahm J Voit D Simion SH Merkenschlager A . Performance of fast and ultrafast T2-weighted MRI sequences for common cerebral lesions in children. Rofo. (2025) 197:526–32. doi: 10.1055/a-2404-8674. Wertigkeit von schnellen und ultraschnellen T2-gewichteten MRT-Sequenzen bei häufigen zerebralen Läsionen bei Kindern., PMID: 39477213

[21] Naldemir İF Karaman AK Ogul H Onbas O . Visual assessment of cerebrospinal fluid flow dynamics using 3D T2-weighted SPACE sequence-based classification system. Acta Radiol. (2024) 65:1576–82. doi: 10.1177/02841851241288219, PMID: 39491809

[22] Teping F Oertel J . Considerations on surgical strategies and associated risk profiles for endoscopic tumor biopsies within the third ventricle and periaqueductal region. Childs Nerv Syst. (2023) 39:3407–14. doi: 10.1007/s00381-023-06122-9, PMID: 37682304 PMC10684420

[23] Sellin JN Cherian J Barry JM Ryan SL Luerssen TG Jea A . Utility of computed tomography or magnetic resonance imaging evaluation of ventricular morphology in suspected cerebrospinal fluid shunt malfunction. J Neurosurg Pediatr. (2014) 14:160–6. doi: 10.3171/2014.4.Peds13451, PMID: 24856881

[24] Jakab A Payette K Mazzone L Schauer S Muller CO Kottke R . Emerging magnetic resonance imaging techniques in open spina bifida in utero. Eur Radiol Exp. (2021) 5:23. doi: 10.1186/s41747-021-00219-z, PMID: 34136989 PMC8209133

[25] Beaumont TL Limbrick DD Patel B Chicoine MR Rich KM Dacey RG . Surgical management of colloid cysts of the third ventricle: a single-institution comparison of endoscopic and microsurgical resection. J Neurosurg. (2022) 137:905–13. doi: 10.3171/2021.11.Jns211317, PMID: 35148502

[26] Unal TC Sencer A Dolas I Gulsever CI Sahin D Dolen D . Full-endoscopic removal of third ventricular colloid cysts: technique, results, and limitations. Front Surg. (2023) 10:1174144. doi: 10.3389/fsurg.2023.1174144, PMID: 37334201 PMC10272465

[27] Fukuhara N Nishihara T Sato K Inoshita N Tatsushima K Yamaguchi-Okada M . Long-term outcomes of neuroendoscopic cyst partial resection combined with stereotactic radiotherapy for craniopharyngioma. Acta Neurochir (Wien). (2024) 166:218. doi: 10.1007/s00701-024-06113-y, PMID: 38750340

[28] Adebayo BO Kanu OO Bankole OB Ojo OA Adetunmbi B Morgan E . Early outcome of endoscopic third ventriculostomy with choroid plexus cauterization versus ventriculoperitoneal shunt as primary treatment of hydrocephalus in children with myelomeningocele: A prospective cohort study. Oper Neurosurg. (2021) 21:461–6. doi: 10.1093/ons/opab314, PMID: 34662909

[29] Zhou J Zhong Y Li X Li H Wang J Yang S . Risk factors for external ventricular drainage-related infection: A systematic review and meta-analysis. Neurol Clin Pract. (2023) 13:e200156. doi: 10.1212/cpj.0000000000200156, PMID: 37529300 PMC10238084

[30] Munoz-Gualan AP Bektasoglu PK Yamaner EO Gungor A Ture U . The extreme anterior interhemispheric transcallosal approach for pure aqueduct tumors: an anatomical study. Turk Neurosurg. (2023) 33:840–6. doi: 10.5137/1019-5149.Jtn.42429-22.3, PMID: 37528717

[31] Ishikawa T Takeuchi K Tsukamoto N Kawabata T Wakabayashi T . A novel dissection method using a flexible neuroendoscope for resection of tumors around the aqueduct of sylvius. World Neurosurg. (2018) 110:391–6. doi: 10.1016/j.wneu.2017.11.044, PMID: 29158099

[32] Li Q Dong J Yang Q Yang H Luo X . Pineal germinoma diagnosed by iohexol CT ventriculography. Pediatr Radiol. (2025) 55(2):359. doi: 10.1007/s00247-024-06120-2, PMID: 39652130

